# The ELUCID Study: Identifying mechanisms and risk factors for dementia in individuals with late‐onset unexplained epilepsy

**DOI:** 10.1002/alz70856_097236

**Published:** 2025-12-24

**Authors:** Kyle R Pellerin, Tania Anderson, Maria Camitan, Alexis Hankerson, Aseel Jan, Katie Jung, Maddie Leake, Timothy O'Brien, Madeline Taubkin, Rebecca E. Amariglio, Leah Blank, Tyler Gaston, Emily Johnson, Rani A Sarkis, Mouhsin Shafi, Rodrigo Zepeda, M. Brandon Westover, Alice D Lam, ELUCID Study Group

**Affiliations:** ^1^ Massachusetts General Hospital, Boston, MA, USA; ^2^ University of Alabama Birmingham, Birmingham, AL, USA; ^3^ Johns Hopkins Medical Institute, Baltimore, MD, USA; ^4^ Brigham and Women's Hospital, Boston, MA, USA; ^5^ University of Texas Southwestern, Dallas, TX, USA; ^6^ Icahn School of Medicine at Mount Sinai, New York, NY, USA; ^7^ Beth Israel Deaconess Medical Center, Boston, MA, USA; ^8^ Harvard Medical School, Boston, MA, USA

## Abstract

**Background:**

Late‐onset unexplained epilepsy (LoUE), defined as epilepsy starting after age 55 with no clearly identified cause, has emerged as a significant risk factor for dementia. Individuals presenting with LoUE have no prior history of dementia. Yet, LoUE is associated with a 2‐3x increased risk of developing dementia, and up to 25% of individuals with LoUE develop dementia within 4 years after their first seizure. We have little understanding of the mechanisms that underlie development of dementia in LoUE.

**Method:**

The ELUCID Study (Epilepsy of Late‐onset Unknown etiology as a risk factor for Cognitive Impairment and Dementia) is a multi‐center, prospective longitudinal observational study of LoUE, focused on understanding mechanisms and predicting outcomes of mild cognitive impairment and dementia in LoUE. ELUCID will enroll 600 participants with LoUE (and without dementia) across 7 study sites. Participants undergo a baseline evaluation with clinical history, cognitive testing, brain MRI, overnight scalp EEG, and blood draw, and are followed longitudinally with interval history every 6 months and annual cognitive testing. The primary outcomes are development of mild cognitive impairment and dementia.

**Result:**

To date, 67 ELUCID participants have completed their initial study visit, with mean age of 67.9±7.2 years and 38.8% female. The sample includes 89.6% White, 3% Black, 1.5% Asian, 6% unreported race, and 1.5% Hispanic ethnicity. Mean level of education was 16.9±2.7 years. Vascular risk factors were common, including hypertension (51%), hyperlipidemia (58%), diabetes mellitus (6%), coronary artery disease (9%), and obstructive sleep apnea (28%). A family history of seizures was present in 23.9% of participants, and a family history of dementia in 58%. Cognitive test scores largely fell within normal range, including: MMSE: 28.7±1.5; Logical Memory Delayed: 11.9±3.4; FCSRT Free Recall: 31.6±6.4; Trails B: 94.3±54.4; Digit Symbol Substitution: 41.9±10.1; and Category Fluency (animals): 17.0±4.9. Subjectively, 32.8% of participants felt their memory had worsened compared to 6 months prior.

**Conclusion:**

The ELUCID Study is a large longitudinal study of LoUE that will define its relationship to Alzheimer's disease and related dementias. Here we describe the study protocol and provide an early report of the baseline demographic and clinical characteristics of the accruing ELUCID study population.

